# Segmental Darier’s Disease Treated With Cryotherapy

**DOI:** 10.7759/cureus.72068

**Published:** 2024-10-21

**Authors:** Ngoc Nguyen, Kevin Liu, Marvin Sasson, Kirsten Bellucci

**Affiliations:** 1 Medical Education, Philadelphia College of Osteopathic Medicine, Philadelphia, USA; 2 Dermatology, Lehigh Valley Health Network, Allentown, USA

**Keywords:** atp2a2 gene, cryotherapy, darier’s disease, keratotic papules, segmental darier's disease

## Abstract

Darier's disease is characterized by pruritic keratotic papules resulting from dysfunctional keratinocyte adhesion. Segmental Darier's is a rare variant thought to be caused by a post-zygotic somatic mutation. The mainstay of treatment consists of oral and topical retinoids and corticosteroids. Other treatments include topical calcineurin inhibitors, 5-fluorouracil, vitamin D analogs, ablative lasers, and surgical excision in refractory cases. Segmental Darier's may be difficult to recognize and even more challenging to treat. Herein, we present a case of a 45-year-old female with linear segmental Darier's disease who improved significantly following treatment with cryotherapy.

## Introduction

Darier's disease is a genetic acantholytic disorder that clinically presents as keratotic papules on sebaceous and intertriginous surfaces. It is inherited in an autosomal dominant pattern due to a mutation in the ATP2A2 gene, which plays a role in regulating calcium channels in the endoplasmic reticulum. Loss of function in its transcription product, sarcoplasmic/endoplasmic reticulum Ca2+-ATPase 2 (SERCA2), results in dysfunctional keratinocyte adhesion [1]. Somatic mosaicism can result in a segmental form of Darier's disease, with about 10% of Darier's cases presenting as a localized disease [1,2]. Treatment typically involves oral and topical retinoids and corticosteroids. We herein report a case of segmental Darier's disease treated with liquid nitrogen cryotherapy.

## Case presentation

**Figure 1:**
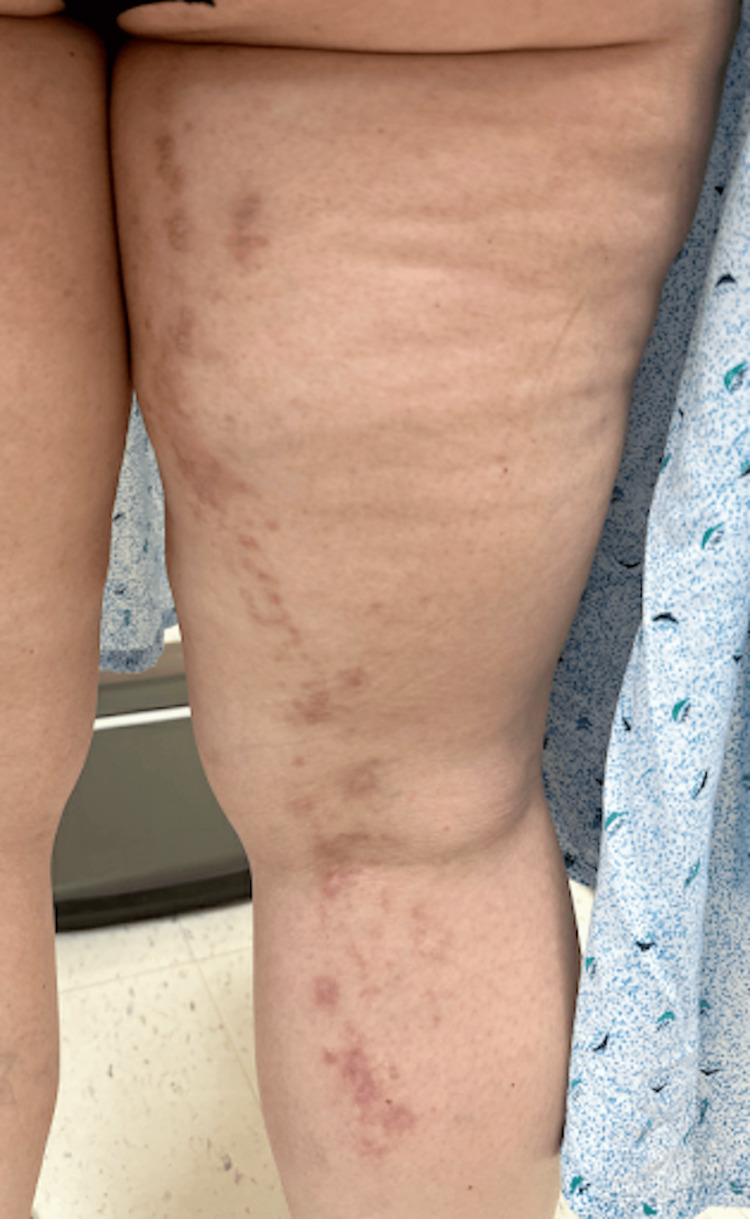
Pink keratotic papules in a linear distribution on the right posterior thigh and leg

**Figure 2:**
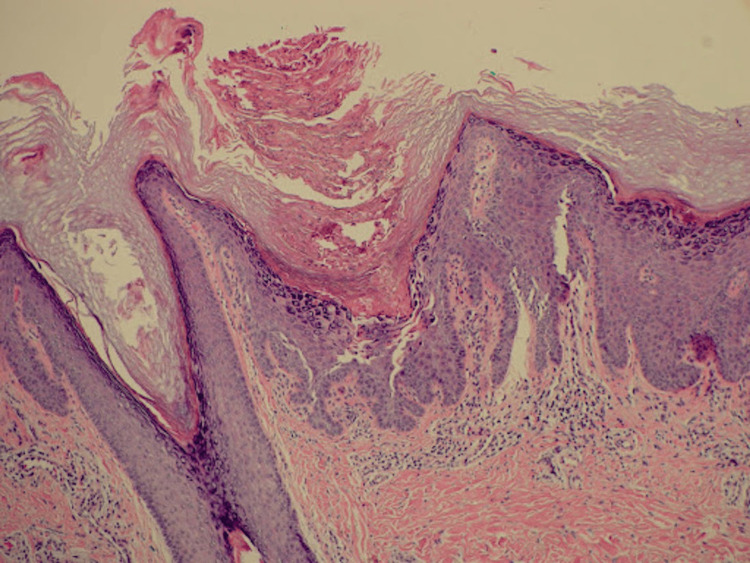
Punch biopsy demonstrates focal suprabasilar acantholysis and dyskeratosis, consistent with Darier’s disease

**Figure 3:**
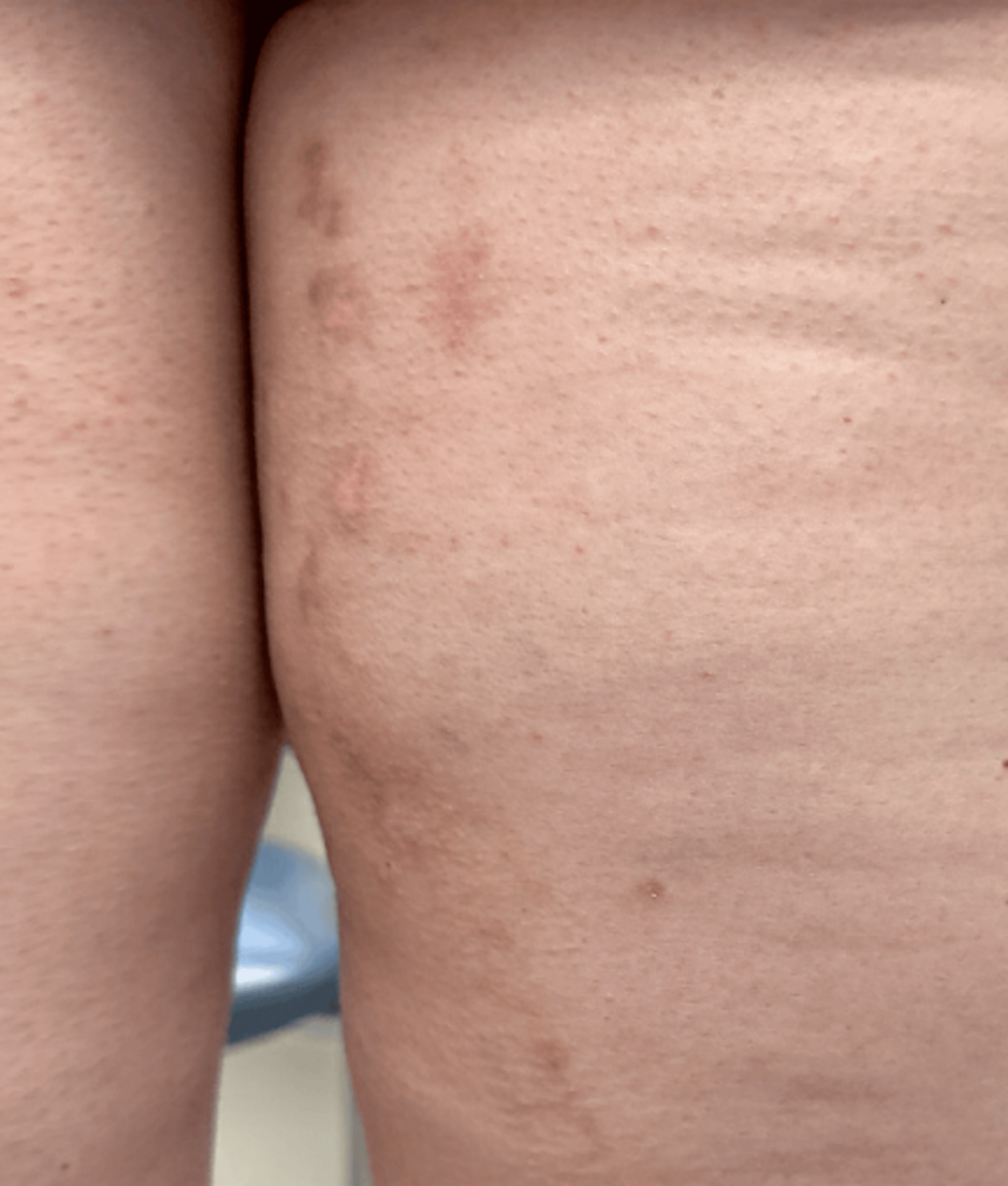
Treatment with cryotherapy results in flattening of the skin texture with post-inflammatory erythema

A 45-year-old female patient presents with a rash on her right posterior lower extremity. The rash first developed about 15 years prior on the distal thigh and popliteal fossa and has since spread proximally. The lesions are sometimes pruritic, but otherwise, they are asymptomatic. The rash waxes and wanes and is notably worse with stress and exposure to sunlight. She has also noticed that the rash transiently improves with short courses of prednisone. Her medical history is only significant for hyperlipidemia and hypothyroidism. She was seen by an outside dermatologist 10 years ago and was treated with imiquimod and cryotherapy under a presumed diagnosis of verruca. The application of imiquimod exacerbated the rash, but the lesions resolved with liquid nitrogen cryotherapy, and the patient had remained clear for several years before the rash returned. On exam, there are pink keratotic papules in a somewhat linear distribution on the right posterior thigh and leg (Figure 1). Examination of the nails and oral mucosa demonstrates no abnormal findings. The patient is empirically treated with triamcinolone 0.1% cream for possible lichen striatus without improvement. At follow-up two months later, due to lack of improvement, the patient is switched to clobetasol 0.05% cream, and a punch specimen is obtained for pathology. Histology of a characteristic lesion shows papillomatous epidermal hyperplasia with acantholytic dyskeratosis (Figure 2), consistent with a diagnosis of segmental Darier’s disease. Given the patient’s success with cryotherapy in the past, she was treated once again with this method. She was also started on tazarotene 0.1% cream with an improvement of her rash from the combination of treatments (Figure 3).

## Discussion

Darier's disease, also known as keratosis follicularis, is characterized by keratotic papules due to dysfunctional keratinocyte adhesion, resulting in acantholysis and dyskeratosis. Up to 10% of Darier's cases present as the segmental variant and are thought to be caused by post-zygotic somatic mutations in the ATP2A2 gene [2]. The ATP2A2 gene plays a role in intracellular adhesion, and mutation of this gene results in disruption of the normal epidermal architecture. Histologically, Darier’s disease shows these features of abnormal cell adhesion and maturation with acantholysis resulting in suprabasal clefts and dyskeratosis with corp ronds and grains [3]. Both the generalized and segmental forms show similar histological findings [1]. 

Generalized Darier’s disease presents with pruritic, symmetrical keratotic yellow-to-brown papules and plaques. These lesions typically appear in intertriginous areas and seborrheic areas of the trunk, scalp, and forehead [1,2]. Less frequently seen are keratotic papules on the palms and soles and subungual keratosis. Nail abnormalities are common and include a characteristic V-shaped notching due to nail fragility, with alternating white and red longitudinal streaks. Mucocutaneous involvement is also typical in generalized Darier’s disease, manifesting as hypochromic papules and cobblestoning of the hard palate, tongue, and gingivae [2-4].

While the classic form presents with widespread skin lesions, segmental Darier's disease is characterized by localized lesions. The segmental variant tends to present later in life, and these patients do not have mucosal or nail involvement. There are two phenotypes of the segmental variant. Type I is characterized by unilateral, localized lesions in a Blaschkoid distribution, and Type II is characterized by a generalized distribution with focal areas of increased severity [2]. If genetic mosaicism of the localized disease affects the gonads, offspring may inherit the generalized disease [1]. 

Symptomatic management of segmental Darier's disease includes behavioral modifications and avoidance of triggers. Patients are encouraged to use sun protection, avoid excessive heat, and wear loose-fitting clothing to minimize friction [2,4]. First-line treatment typically includes topical retinoids such as isotretinoin, adapalene, and tazarotene, which modulate keratinocyte proliferation [3,5]. Systemic retinoids are also used but carry higher risks of toxicity and adverse effects compared to topical formulations [3]. Oral and topical corticosteroids are used for their anti-inflammatory effects to help alleviate pruritus and irritation [1]. Additional topical therapies such as 5-fluorouracil, calcineurin inhibitors, and vitamin D analogs have shown varying degrees of success in the literature [5]. Adjunctive treatments with dermabrasion, ablative lasers, and excision have also been documented [1]. To our knowledge, cryotherapy with liquid nitrogen has not been reported as a treatment for Darier's disease. 

Our patient presented with the Type I form of segmental Darier's disease that did not improve with imiquimod or high-potency topical steroids. Treatment with liquid nitrogen and tazarotene cream resulted in improvement of the rash. The patient tolerated this combination therapy, which resulted in significant clinical improvement, highlighting a promising novel therapeutic approach for segmental Darier's disease. 

## Conclusions

Segmental Darier's disease is a rare variant of Darier's characterized by localized skin involvement. The diagnosis is challenging and should be considered in cases of a unilateral rash that demonstrates acantholytic keratosis on histology. Management may be complex, and treatment options have varying degrees of success. Our case report demonstrates successful treatment of localized Darier's disease with cryotherapy, suggesting that this modality may be considered as a therapeutic option, especially in cases refractory to conventional therapies. Cryotherapy can be a fast and affordable treatment for this condition, but patients should be aware of the potential of dyspigmentation. Further studies are needed to evaluate the long-term efficacy and effects of cryotherapy in the treatment of localized Darier's disease. 
